# Influencing factors and optimization paths of teachers’ fulfillment of school bullying governance responsibilities in China

**DOI:** 10.1371/journal.pone.0319640

**Published:** 2025-12-05

**Authors:** Fuyu Li, Xiaohu Yang, Shifang Meng, Haibo Zhang

**Affiliations:** 1 Criminal Investigation and Counter-Terrorism College, Research Center of National Security Governance, Criminal Investigation Police University of China, Shenyang, Liaoning, China; 2 School of Marxism, Shihezi University, Shihezi, Xinjiang, China; 3 School of Humanities and Law, Northeastern University, Shenyang, Liaoning, China; 4 School of Marxism, Wenzhou University, Wenzhou, Zhejiang, China; Universiti Pertahanan Nasional Malaysia, MALAYSIA

## Abstract

The effective fulfillment of teachers’ responsibilities is crucial to the governance of school bullying. Guided by Grounded Theory and utilizing NVivo11 qualitative analysis software, this paper used 316 texts from 55 cases of school bullying in China to conduct open coding, spindle coding and selective coding, analyzed the behavior of teachers, explored the impetus factors and resistance factors influencing teachers’ fulfillment of their responsibilities, and constructed a “two-factors model”. The reasons why these factors in the model affect school bullying governance responsibility of teachers were analyzed through Street-Level Bureaucracy Theory. On the one hand, teachers are legally obligated to provide fair and equitable educational services to students. The media and the public are important forces in monitoring teachers’ fulfillment of their responsibilities. Additionally, leaders from school and the local government such as police department utilize interdependency and gaming relationships with teachers to promote teachers’ fulfillment of their responsibilities. Furthermore, the formulation of laws and policies is an important basis for supervising teachers’ behavior. These factors collectively promote teachers’ fulfillment of school bullying governance responsibilities. On the other hand, teachers have discretion and are difficult to supervise and hold accountable. The interests of different governance subjects may diverge or conflict. Moreover, the complexity and diversity of school bullying exacerbate governance challenges. Furthermore, systems such as laws and policies often fall short in restraining undesirable behavior. These reasons present obstacles to teachers’ fulfilling their responsibilities of school bullying governance. In order to strengthen the impetus factors for teachers to fulfill their responsibilities and mitigate resistance, China needs to optimize the governance path. This involves improving the relevant systems, enhancing publicity, providing education and training for teachers, strengthening the supervision of the local government, the media and the public on teachers, and improving the overall governance environment for school bullying. These measures will encourage teachers to hold themselves to higher standards and effectively fulfill their responsibilities in the governance process of school bullying.

## 1 Introduction

Including China, and serious school bullying is detrimental to the development of a country. According to the “School Violence and Bullying Global Status Report” released by United Nations Educational, Scientific and Cultural Organization (UNESCO) in 2017, nearly 246 million students worldwide had been bullied in schools [1]. In 2019, statistics from the National Center for Education Statistics in America showed that about 1 in 5 students had been bullied in school and 41% percent of students believed that they were at risk of being bullied again [2]. In the opinion of the Danish scholar Dan Olweus, a pioneer in the international study of school bullying, school bullying refers to the humiliating physical and mental attacks occurring between individuals and groups of students with unequal power in the school and its surrounding areas, which results in mental or physical harm inflicted upon the bullied [3]. School bullying is characterized by intentionality, repetition, anonymity, and difficulty in disclosure [4]. School bullying has a negative impact on the physical, psychological, and behavioral aspects of students, especially those who are bullied. Different forms of school bullying, such as physical, relational, verbal, and sexual bullying, can result in different harmful consequences [5]. School bullying is extremely harmful to the physical health of victims, resulting in their physical pain, or leading to the physical disability and even death of the bullied. It is strongly associated with the increased rates of self-harm among students [6]. It also affects the well-being of students, especially victims [7]. Victims may have psychological problems such as anxiety, depression, and low self-esteem [8], and are more likely to generate suicidal thoughts [9]. The adverse consequences for young people who experience bullying during their school years include mental and physical health problems as well as body image issues, diet disorders, social anxiety and other issues [10]. Existing research suggests that there is a link between school bullying and sleep disorders in some adolescents [11]. Besides, school bullying seriously jeopardizes the high quality and sustainability of education. This is due to the relatively low classroom participation of victims [12]. They lack a sense of security in school, have trouble concentrating while studying, experience declining grades, and resist going to school [13]. Compared with their peers, victims of bullying face an increased risk of experiencing declining academic performance [14], and in severe cases, they have to drop out of school or even embark on a path toward delinquency [15]. Therefore, school bullying seriously harms the academic development of the bullied and is not conducive to the sustainability of education.

In China, the problem of school bullying also exists. According to a 2019 survey report named “Behind the Numbers: Ending School Violence and Bullying” from UNESCO, nearly one-third of children globally still suffered from school bullying every year. Among them, the incidence of school bullying in four cities in Chinese mainland was 20.2% in Beijing, 31.8% in Hangzhou, 31.9% in Urumqi, and 33.2% in Wuhan [16]. In the face of ongoing incidents of school bullying, China promulgated “Notice of the Education Supervisory Committee of the State Council on Carrying out a Special Governance Against School Bullying” in 2016, which is the first policy document at the national level in China that governs school bullying as a specialized problem [17]. The policy clarifies the governance responsibilities of schools and teachers. Police and other relevant departments carry out legal education in schools. In the same year, China enacted “Guiding Opinions of Nine Departments Including the Ministry of Education on Preventing and Disposing Bullying and Violence Among Primary and Secondary School Students”, which stipulates that school principal is the primary person responsible for preventing and controlling school bullying, and that the vice-principal of rule of law education and the class teacher are directly responsible for these matters. Government departments such as education and police should protect the victims of school bullying, handle cases involving school bullying in accordance with the law, and strengthen cooperation among various departments. In 2017, the “Program for Strengthening Comprehensive Governance of Bullying Among Primary and Secondary School Students”, issued by 11 departments, including the Ministry of Education, explicitly states that the disposal of school bullying is to be carried out mainly by schools, with teachers being accountable for the specific implementation of the work. Eleven departments, including education and police, work together to strengthen the governance of school bullying among primary and secondary schools. Law on the Protection of Minors of the People’s Republic of China, revised in 2020, explicitly mandates that teachers set up a system for preventing and controlling school bullying, carry out education and training, discipline bullies in accordance with the law, and deal with incidents of bullying in time. Regulations on the Protection of Minors at Schools, promulgated in 2021, extensively outline the responsibilities of teachers, such as prevention, correction, timely investigation and intervention, as well as educating, disciplining or punishing bullies. Police and other departments cooperate with schools in the investigation, prevention and disposal of school bullying. However, despite China’s revision of laws, promulgation of policies, and adoption of measures, school bullying continues to occur. In 2022, Hua Long et al. conducted a sample survey on school bullying in some regions of China, and the result showed that 39.59% of the bullied were detected while 14.46% of the bullies were reported [18]. On June 1, 2023, the Supreme People’s Procuratorate released the White Paper on Juvenile Procuratorial Work (2022), which revealed the number of approved arrests and prosecutions of bullying and violence criminals from 2020 to 2022 [19]. The specific data are shown in Table 1. In fact, more incidents of school bullying go unnoticed, and more bullies are neither arrested nor prosecuted. The situation of school bullying governance in China remains a cause for concern.

**Table 1:** Number of people arrested or prosecuted for school bullying and violent crimes in China from 2020 to 2022.

Year	Number of Approved Arrests	Number of Prosecutions	Sum
2020	583	1341	1924
2021	581	1062	1643
2022	271	684	955

School bullying mainly takes place in schools, including kindergartens, primary schools, junior high schools, senior high schools and colleges. It is commonly seen in primary and secondary schools [20], and junior high schools are the most frequent settings for such incidents [21]. This characteristic is reflected in the forms of bullying such as cyberbullying [22]. Moreover, many laws and policies addressing school bullying are aimed at tackling bullying in primary and secondary schools. As such, it is crucial that teachers in the schools, especially those in the primary and secondary schools, conscientiously fulfill their responsibilities in order to effectively achieve the goal of school bullying governance. In this paper, the teachers refer to class teachers, head teachers, life guidance teachers, psychology teachers, dormitory administrators and other teachers who directly contact with students and parents. On the basis of sorting out typical cases of school bullying and combined with domestic and foreign literature on teachers’ fulfillment of responsibility, our research provides recommendations for teachers to optimize behavior and better fulfil their responsibilities in tackling school bullying.

## 2 Literature review

The fulfillment of the responsibility of school bullying governance is a widely influential research topic, and the current research findings on teachers’ fulfillment of the responsibility of bullying governance are mainly explored from the following perspectives.

Firstly, many countries have enacted laws and policies on school bullying governance, and scholars have sorted out the liability requirements put forth therein for teachers. In the United States, schools are required by strict legislation to punish school bullying. Once school bullying is detected, measures must be taken immediately, which can deter bullies through severe punishments such as corporal punishment, class suspension, expulsion, or even referral to juvenile justice institutions [23]. Although the content of school bullying governance laws varies from state to state, they all treat teachers as the main body of governance, with teachers assuming responsibility for reducing school bullying [24]. In Norway, the government works with the National Teachers’ Association, anti-bullying coalitions in different regions and other entities to implement school bullying governance policies and zero-tolerance programs [25]. In order to curb the problem of school bullying, the UK government has passed legislation to provide schools with a legal foundation for school bullying governance. It has also offered pre-service and on-the-job training for teachers [26], and strengthened governance of school bullying by giving teachers more disciplinary powers and responsibilities [27]. For the sake of combating school bullying, China has issued a series of policy documents that clarify the responsibilities of multiple subjects while emphasizing the responsibilities of teachers [28]. Regarding views on the governance of school bullying, many teachers focus on individual responsibility rather than social or systemic responsibility [29]. For some special students, such as those diagnosed with autism spectrum disorder (ASD) or intellectual disability(ID), teachers should undertake the responsibility of protecting them from peer bullying [30].

Secondly, scholars have explored the types of responsibilities that teachers need to assume. When it comes to the governance of school bullying, teachers have to be held accountable for their own behavior and reduce bullying through anti-bullying policies and other measures [31]. Teachers need also to be held liable for any negligence in taking the adequate measures to prevent school bullying. Should a student suffer harm because of bullying, the school shall bear civil tort liability and non-joint liability [32]. As schools play an important role in the governance of bullying, the responsibilities of principals [33] and teachers [34] have become the focus of research. Principals and teachers have an obligation to proactively safeguard students from mental or physical bullying, and a person who violates the obligation will be subject to vicarious liability for fault [35]. If the consequences of school bullying are serious, schools and teachers should bear administrative, civil or criminal responsibilities [36]. A safe school environment is a protective element, and responsible teachers reduce the risk of school bullying by providing supportive, supervisory behavior [37].

Thirdly, scholars have explored some of the factors that affect the fulfillment of teachers’ responsibilities. Because of their unique status as important implementers of school bullying governance, teachers have been entrusted with more responsibilities as the most central and possibly the most effective governance subjects [38]. However, not all teachers are able to fulfill their responsibilities conscientiously, or may wish to do so but have difficulty in achieving the desired governance goals. Therefore, it is necessary to explore the factors influencing teachers’ fulfilling the responsibility of bullying governance. Researchers have found that due to the constraints of current laws and policies, the duties of governance subjects are set in general terms and the boundaries are unclear, which is not conducive to the fulfillment of teachers’ governance responsibilities [39]. Teachers are responsible for addressing school bullying among students, and their willingness to fulfill responsibilities depends on their knowledge, attitudes, sense of responsibility, and confidence in dealing with school bullying [40]. Preservice teachers will one day be responsible for addressing school bullying among their students. But few of them understand the hallmarks of school bullying, and many do not feel confident in their ability to deal with school bullying [40]. Bias-based bullying of sexual and gender minority often is pervasive in hostile school environment. In order to avoid this kind of school bullying, it is very necessary to establish a positive school climate. Participants are recommended multilateral interventions and training, including teacher-student and teacher-administrator interactions [41].Some teachers have deviated from their roles and have improper cognition of some policy contents, thus hindering the effective fulfillment of governance responsibilities [42]. Additionally, some teachers have insufficient understanding of their roles in school bullying governance and exhibit weak motivation to construct roles, limiting their fulfillment of responsibilities [43].

Scholars have discussed the institutional requirements, types of behavior responsibilities and some influencing factors for teachers to fulfill their responsibilities of school bullying governance. These academic results have laid a certain foundation for the subsequent research, but the current research results still have room for improvement. Many scholars studying teachers’ fulfillment of governance responsibilities either put schools together with the government, families and other governance subjects to discuss the common factors affecting them, without delving into the factors affecting teachers’ fulfillment of their governance responsibilities in depth and in detail; or explore the responsibilities that teachers need to fulfill and their impacts from the perspective of objective factors such as law, which is a relatively narrow perspective. At present, there are many academic achievements on teachers’ fulfilling the responsibility of school bullying governance, but few studies discuss teachers’ behavior from the perspective of street-level bureaucrats to strengthen their fulfillment of responsibility. Existing research that explores the fulfillment of teachers’ responsibilities in the governance of school bullying either analyses teachers as a part of school when discussing the school’s responsibilities, or explores teachers as a group as a whole. As a group, teachers are at different levels and responsible for different work. In some primary and secondary schools, principals teach classes and are also involved in school management, playing the dual role of principal and teacher. There are also teachers who are only responsible for administrative work and do not have direct contact with students and parents. Teachers at different levels are in different positions, playing different roles, performing different functions and fulfilling different duties. Grassroots teachers interact directly with students and parents, and have a better understanding of school bullying situation. They are the key players in the governance of school bullying, and the fulfilment of their responsibilities is very important to the effective governance of school bullying. Therefore, this paper focuses on the role of grassroots teachers in school bullying governance, and explores the influencing factors of teachers’ fulfilment of their responsibilities based on the Theory of Street-Level Bureaucracy.

**Fig 1:**
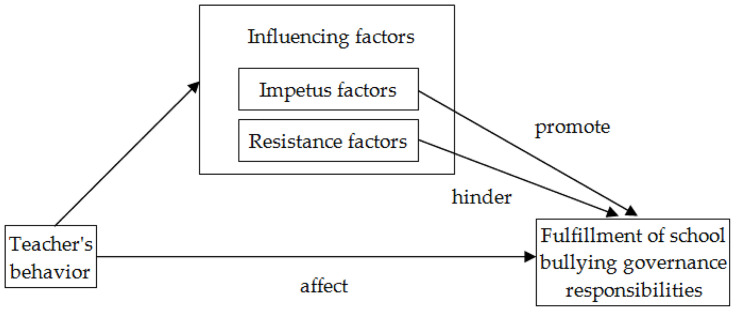
The research idea of this paper.

In the process of school bullying governance, are there any other factors affecting teachers’ fulfillment of governance responsibilities besides laws and policies? What are the reasons for the varying degrees to which different teacher fulfill their responsibilities in the process of school bullying governance? This paper selected 55 bullying incidents in the primary and secondary schools as the object of analysis, analyzed in depth the performance of teachers’ fulfillment of responsibilities in the cases with the help of NVivo11 software, and tried to explore two questions: What factors influence the behavior of teachers in fulfilling their responsibilities in the process of school bullying governance? What measures can effectively promote teachers to fulfill their responsibilities in school bullying governance? Then this paper extrapolated the reasons for teachers’ failure to fulfill their responsibilities based on the Theory of Street-Level Bureaucracy, and put forward optimization suggestions. The research idea of this paper is shown in Fig 1.

## 3 Research method and theory

### 3.1 Research method

Case studies can be used to explain hypothetical causal relationships assumed to exist between different factors in reality that are difficult to study through experiments or surveys, or to explore less obvious and complex and variable causal links [44]. Multiple case studies can overcome the drawbacks of single case studies such as the problem of generalizability of conclusions and the insufficiency of extrinsic validity [45]. Compared with single case studies, multiple case studies are more persuasive and stand up to scrutiny, and cross-case conclusions drawn from case-crossover analysis have broader applicability.

### 3.2 Grounded theory and NVivo qualitative analysis software

Qualitative data will produce meaningful findings when they are managed properly. Grounded Theory is a qualitative analysis method proposed by Barney G. Glaser and Anselm L. Strauss in the book named *The Discovery of Grounded Theory*, which collects qualitative materials and a complete series of data coding processes to form a set of unique insights on a certain issue and conduct theoretical reconstruction. NVivo software is a software for qualitative data analysis that applies the ideas of Grounded Theory to conduct open coding, spindle coding and selective coding for qualitative material. It is a program created by QSR International, its first version released in 1997 [46]. Later on, NVivo software has been upgraded and its functions have been expanded continuously. NVivo11 software can be used to analyze many types of data, such as texts, pictures, audios, videos and so on. In this paper, 55 typical cases of school bullying in China in recent years were used as research samples, and the NVivo11 qualitative analysis software was applied to analyze the content and summarize code the data of 316 case textual materials. Then the paper explored the issue of teachers’ ineffective fulfillment of their responsibilities in managing school bullying through textual content analysis.

### 3.3 Theory of street-level bureaucracy

In 1980, the American scholar Michael Lipsky’s masterpiece *Street-Level Bureaucracy: Dilemmas of the Individual in Public Services* came out, marking the formal establishment of the Theory of Street-Level Bureaucracy. In the theory, street-level bureaucrats are public service workers who directly interact with citizens in the process of executing their work and have a great deal of discretion, and typical street-level bureaucrats include teachers, police officers, judges, public lawyers, health workers, and other public employees [47]. Street-level bureaucrats exist at the bottom of public service sectors such as education, police, courts, customs, social welfare, etc., and the grassroots units of these departments are called street-level bureaucracies. The main views of the Street-Level Bureaucracy Theory include the following aspects.

Street-level bureaucrats provide public goods or services in their interactions with the public. They interact directly with the public in the process of governance, provide public goods or services to the public, and make decisions for individuals on the spot, which are closely related to the public’s immediate rights and interests. The interaction may take place on the street, in a street-level bureaucracy, or even in the homes of some street-level bureaucrats. Street-level bureaucrats may adopt some strategies to reduce the willingness of the public to seek services, and may treat the public with favoritism and discrimination. Citizens can appeal if they encounter unfair treatment or give feedback through evaluation mechanisms.

Street-level bureaucrats wield significant discretion in determining the nature, quantity, and quality of the services and sanctions they provide. This discretion arises from the ever-changing nature of their work environments, where they must make quick decisions due to limited resources such as time and information. Having discretion is one of the salient characteristics of street-level bureaucrats, which may be a result of administrative or legal authority, or the dominance formed due to some kind of resource advantage. The decisions of street-level bureaucrats, the practices they establish and the means they invent to cope with the pressure of their work will all become the new policies they implement.

Street-level bureaucrats and superior managers are interdependent and engage in a game. They are interdependent because superior managers have disposable funds, master the authority of position promotion, work assignments and so on, while street-level bureaucrats depend on the support of superior managers to accomplish their jobs and career development. Superior managers also rely on street-level bureaucrats to receive and execute the work, and street-level bureaucrats have the advantage of information and specialization. The existence of the game between the two sides stems from the differences in their needs and positions, which lead to differences in goals, interests, and perceptions. For example, street-level bureaucrats want to maintain and expand discretion, while managers try to limit it.

Supervision and accountability can constrain street-level bureaucrats, but often yield minimal impact. Achieving accountability among the grass-roots personnel who exercise a high degree of discretion is difficult, particularly with respect to the quality of work. Decisions of street-level bureaucrats are made privately or beyond supervision, rendering it difficult to second-guess and hold them accountable for the appropriateness of their decisions. Moreover, the professional nature of some street-level bureaucrats’ work complicates accountability efforts. Implementing accountability measures may lead to a deterioration in the quality of services, and street-level bureaucracies rely on street-level bureaucrats and may refrain from excessive control for fear of objections. With the development of the society, the public expects more and better public services, which inevitably increases the work pressure on street-level bureaucrats. Increased workload will reduce the work quality of street-level bureaucrats, heighten their discretion and make supervision more difficult.

With the development of the society, the content of the Theory of Street-Level Bureaucracy is changing. Unlike Lipsky, who described street-level bureaucrats as passive, Moore saw street-level bureaucrats as positive, creative and goal-seeking politicians [48]. With the application of computers and the Internet, many decisions made by street-level bureaucrats are now carried out by software programs, resulting in a narrowed scope of discretion. System designers have emerged as a new type of street-level bureaucrat. The government gets certain services by allocating funds to social organizations or enterprises, and the personnel of these institutions have the characteristics of street-level bureaucrats, thus being regarded as a new type of street-level bureaucrats. When social services exhibit the characteristics of decentralization and individuation, Rice advocates the combination of the Theory of Street-Level Bureaucracy with the Theory of Institutionalism to explore a micro-institutionalism theory of policy implementation [49]. Although the research object varies, the Theory of Street-Level Bureaucracy still has positive guiding significance.

Street-Level Bureaucracy Theory is also applicable to the analysis of teachers’ fulfillment of their responsibilities in the governance of school bullying. This is because, according to the content of the Theory of Street-Level Bureaucracy, teachers are typical street bureaucrats in schools, providing education as a public service to students and parents. Teachers interact directly with the students or parents, and have a certain degree of discretion in terms of education quality, caring for students and so on. Therefore, it is reasonable for this paper to analyze the influencing factors of teachers’ fulfillment of responsibilities in the governance of school bullying with the help of Street-Level Bureaucracy Theory.

## 4 Data source

### 4.1 Case collection

China began to govern school bullying as a specialized problem in 2016, so the selected school bullying cases in this paper span from 2016 to the cut-off year of 2023, with the dates generally corresponding to the time of each incident. In instances where school bullying has recurred, the date listed reflects the most recent occurrence. The school bullying cases occurred in different provinces across the country (Taiwan, Hong Kong, and Macau were not studied for the time being). All of these cases have attracted extensive public and media attention because of the irresponsible words and behavior of teachers, which reflect their ineffective fulfillment of their responsibilities, and are somewhat typical and representative cases. Based on research needs, case typicality, sources of information, and the current state of governance, this paper selected 55 cases.

### 4.2 Data source and collection

The materials of school bullying incidents used in this study are sourced from public platforms such as the Internet and newspapers, which are mainly obtained through the following methods:

1. Referencing official platforms to compile incident descriptions and disposal result notifications issued by local government departments and schools through their respective official websites, WeChat public accounts, microblogs, etc.
2. Referring to online resources and collecting the incident materials through official media channels, such as CCTV.com, Xinhuanet.com and people.com.cn, and the four major portal websites, namely, Sina.com, 163.com, Sohu.com, and qq.com.
3. Consulting both the printed and electronic versions of newspapers to collect news reports of the incidents.

In this paper, the collected information was sorted out. In order to avoid data redundancy and eliminate repetitive content, a total of 316 texts of about 196,000 words were obtained, which were then categorized into incident narratives, official notifications, website reports, newspaper news, and other types (Table 2).

**Table 2:** Data collection types of school bullying incidents.

Data Type	Content Introduction	ID	Quantity
**Incident narratives**	Description of the bullying by the victims or the bullies and their family members	S	18
**Official response**	Description or attitudes of the incident by the local governments, education administration departments or schools	G	79
**Website report**	Reports of bullying in the official media or the four major portal websites	W	214
**Newspaper news**	Newspaper articles about school bullying	B	5

In order to better analyze the textual data, each document is numbered according to “case number and type of data and number of data”. For example, “4-W-3” indicates the third material of “website report” in Case 4. The case number is based on the chronological order of bullying incidents, and the number of data is based on the source type number in Table 1. When there are multiple sources of the same type of information, they are sorted according to the Chinese phonetic alphabet of the name and the time of publication. If the source is unique, there is no number for the source number, e.g., “4-S” is the only material in Case 4 that was reported by a family member of the victim of bullying.

### 4.3 Case collection and import

55 school bullying cases selected in this paper are both typical and universal, with remarkable characteristics in terms of time, location, school level, the bullies and the bullied.

#### 4.3.1 Occurrence time.

**Fig 2:**
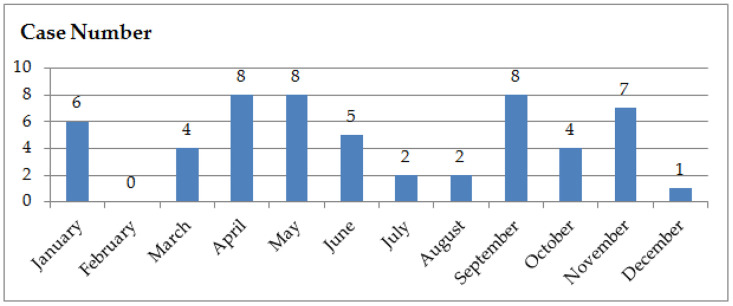
Number of school bullying cases in different months.

The school bullying incidents selected for this paper took place between 2016 and 2023, spanning a period of eight years. There were four cases in 2016, five cases in 2017, nine cases in 2018, 15 cases in 2019, seven cases in 2020, eight cases in 2021, four cases in 2022, and 3 cases in 2023. These cases have certain characteristics in terms of the month of occurrence, with the highest number observed in April, May, and September, followed by November, while February saw no reported cases (Fig 2). This is due to the fact that school bullying mainly happens in schools during regular school hours. In China, February often coincides with the winter vacation period, resulting in fewer incidents during this time. July and August are summer vacations, yet the timing varies across regions, with some schools still having classes in early July and late August, so there are a few incidents of school bullying. Bullying also decreases in months like December when final exams are to be held because students are busy preparing for the exams. There are also fewer incidents when there are festivals or vacations, such as the National Day and Golden Week holidays in October. The cases of school bullying also show a characteristic that school bullying incidents happened in different locations on the same day. For example, Case 23 and Case 24 both occurred on May 17,2019, while Case 41 and Case 42 both happened on January 6, 2021, in Dongguan, Guangdong and Lianjiang, Guangdong. To some extent, this shows that school bullying is prevalent in China.

#### 4.3.2 Occurrence location.

**Fig 3:**
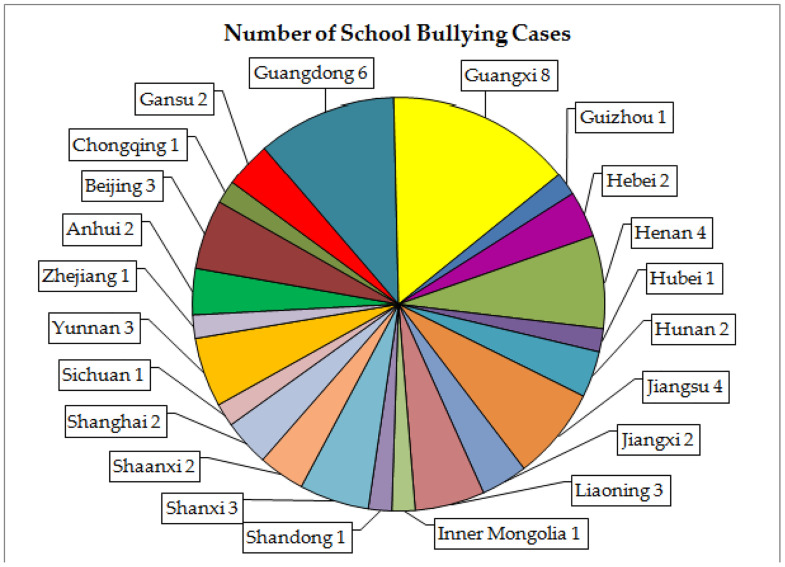
Number of school bullying cases in different provinces.

The school bullying cases selected for this paper occurred in 22 provinces in China (Fig 3), of which Guangxi Zhuang Autonomous Region had the highest number of incidents, totaling 8 incidents. Six incidents happened in Guangdong Province, and four in Jiangsu province and Henan province respectively. School bullying incidents may have occurred multiple times in the same city, as evidenced by Case 7 and Case 12, both occurring in Beihai, Guangxi; Cases 8 and 22 in Shangrao, Jiangxi; Cases 15 and 30 in Xuzhou, Jiangsu; Cases 24 and 27 in Shenzhen, Guangdong; Cases 26 and 45 in Baise, Guangxi; and Cases 36 and 37 in Shenyang, Liaoning. This shows that the failure of teachers’ fulfilling responsibilities is not an isolated case or an occasional occurrence, but rather a common phenomenon in many provinces, and repeated occurrences indicate that they have not been well governed for a long time.

#### 4.3.3 School level.

**Fig 4:**
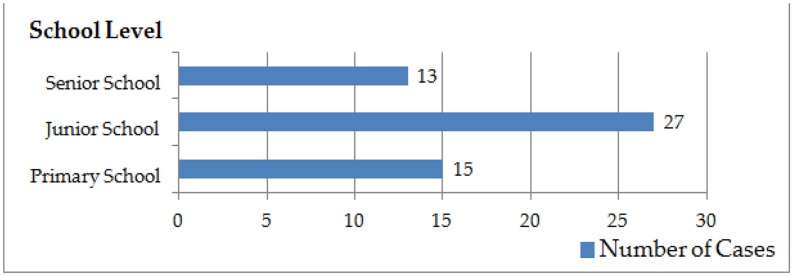
School-level distribution of school bullying cases.

School bullying cases cover three school levels: primary school, junior high school and senior high school. There are 15 cases in primary schools, 27 cases in junior high schools and 13 cases in senior high schools (Fig 4).

#### 4.3.4 The bullies and the victims.

School bullying occurs across different age groups. According to the age divisions of students in different school stages in China, students’ ages were categorized into four stages, namely, 6–12 years old, 13–15 years old, 16–18 years old, and over 18 years old. The distribution of bullies varies across these stages, with 35 bullies aged 6–12, 105 aged 13–15, 33 aged 16–18, and 4 over 18 years old (Table 3). As for the victims, 74 students are 6–12 years old (including 45 in Case 24 and 12 in Case 27), 23 victims are 13–15 years old, 12 victims are 16–18 years old, and 2 victims are over 18 years old (Table 3). According to the age characteristics of the bullies and the victims, it can be found that the age of the bullies is mainly 13–15 years old, and the age of the victims is concentrated in 6–12 years old. The bullies tend to be older than the victims, because in school bullying, older students are more likely to target younger ones. It also proves that bullying occurs more in primary and secondary schools. Besides, school bullying may occur between students of the same gender or between students of different genders. In the 55 cases of school bullying, it was found that out of the total number of bullies, 121 were boys and 57 were girls (Table 3). On the other hand, the number of victims from different genders amounted to 63 boys and 48 girls (Table 3). These gender characteristics reveal that boys are the majority of both bullies and victims in school bullying, indicating that school bullying is more common among boys.

**Table 3:** Characteristics of the bullies and the victims.

		Number of the Bullies	Number of the Victims
**Age**	**6-12**	35	74
	**13-15**	105	23
	**16-18**	33	12
	**>18**	4	2
**Gender**	**Male**	121	63
	**Female**	57	48

The number of the bullies and the victims may vary in different school bullying cases. When the number of students is only one, it is called “single”; while the number is more than one, it is called “multiple”. The comparison of the number of the bullies and the number of the victims falls into the following four categories (the bullies vs. the victims): single vs. single, single vs. multiple, multiple vs. single, multiple vs. multiple. The case numbers in different categories are in Table 4. In the cases of school bullying, the comparison of the number of the bullies and the number of the victims presents a characteristic: There are 43 cases of multiple vs. single, accounting for 78.2% of all cases. This suggests that in China, school bullying often involves multiple students bullying a single person. The bullies may gain psychological advantage through numerical superiority and power imbalance. This also indirectly indicates that the bullied should try to avoid acting alone in their study and life, but rather act together with classmates and friends.

**Table 4:** Characteristics of the number of the bullies and the victims.

Different Categories of the Bullies vs. the Victims	Number of Cases
Single vs. single	6
Single vs. multiple	3
Multiple vs. single	43
Multiple vs. multiple	3

## 5 Coding of the textual data of school bullying cases

In this paper, NVivo11 software is used to analyze the textual data of school bullying incidents. In the first place, 215 textual data of 2/3 of the cases (the first 36 out of 55 cases) were imported into NVivo11 qualitative analysis software, and then open coding, spindle coding and selective coding were carried out to obtain the problems and influencing factors of teachers’ fulfillment of their responsibilities in the process of governance. This coding system was established in the “FY coding” cluster and compared with the second coding “LYY coding” for the reliability test.

### 5.1 Open coding

Open coding is the basis for qualitative analysis of textual data. The process involves reading the data sentence by sentence and importing them into the NVivo11 software. The contents that fit the theme of “Teachers fulfilling responsibilities” were conceptually coded and put into different nodes. A total of 725 nodes were obtained after reading and coding all the textual data, and 67 initial categories were obtained after classifying and integrating all the nodes and categorizing them. The coding rule was “P1 and sequence number”, e.g., P1-1 is the initial category 1 of the open coding (Table 5).

**Table 5:** Open coding of textual material (partial).

Initial Category	Node Numbers	Some Excerpts of Original Material and Sources
**Irresponsible words and deeds of some teachers(P1-1)**	30	①The teacher defined the incident as “just an over-the-top joke.”（4-S）. ②During this time, dormitories 210 and 211 were making particularly loud noises that could be heard clearly in the hallways, but the dormitory administrator ignored them and didn’t care.（21-S）……
**Irresponsible words and deeds of some school leaders(P1-2)**	9	①Principal: “What could possibly be the cause? How much resentment can a third-grader harbor, they’re just fooling around.”（9-W-3）. ②It was only upon receiving the news that the principal rushed to the hospital. In the ward, he pledged on his own behalf and on behalf of the school, promising my family and me a satisfactory answer. But so far, the attitude of the school is one of indifference, with no follow-up. （21-S）……
**Irresponsible words and deeds of some schools (P1-3)**	11	①In response to the recent unfounded statements about our school and related events on the Internet, our school reserves the right to defend the reputation of the students and the school through legal channels and to hold the relevant parties accountable.（4-G-2）. ②Subsequently, the school’s approach has left Jingjing’s parents disheartened. The school failed to proactively investigate whether Jingjing had been subjected to bullying in school before her death. Besides, at the urging of her family, it even acted perfunctorily.（25-W-2）……
**……**	……	……
**Accountability of teachers (P1-66)**	5	①The class teacher of the class in question was punished（10-G). ②On the evening of June 19, following the expanded meeting of the Bureau’s party group, it was decided that the principal, vice principal, political education director, class teacher of Financial Class (36), and dormitory administrator of the vocational high school would be held seriously accountable for their dereliction of duty（21-G-2）……
**Accountability of other duty bearers (P1-67)**	3	①Based on the investigation, the county party committee and the government initiated the accountability procedure and seriously dealt with the relevant responsible persons（9-G-1）.②The next step will also investigate the responsibility and seriously deal with the relevant responsible persons in accordance with the rules and discipline（9-W-1）……

### 5.2 Spindle coding and selective coding

Spindle coding helps to discover the relationship between each node and establish the internal logical connection between different contents of the textual data. Open coding obtained 67 initial categories that were independent of each other. Spindle coding, after clustering and integration of open coding, resulted in 21 subcategories such as “some faculty members’ dereliction of duty in words and deeds”. The rule of spindle coding was “P2 and serial number”, e.g., P2-1 is the first subcategory of spindle coding. All the subcategories were systematically classified into 8 main categories according to their categorical relationships, such as “some schools and staff lack the sense of responsibility”. The coding rule was “P3 and serial number”, e.g., P3-1 indicates the first main category of spindle coding (Table 6). The coding results were put into the cluster “FY coding” to prepare for the coding consistency test.

**Table 6:** Spindle coding and selective coding of textual material.

Main Category	Subcategory	Node Number	Initial Category
**Some schools and staff lack the sense of responsibility** **(P3-1)**	Some faculty members’ dereliction of duty in words and deeds (P2-1)	39	Irresponsible behavior and words of some teachers(P1-1); Irresponsible words and actions of some school leaders(P1-2)
	Some schools’ dereliction of duty in words and deeds (P2-2)	13	Irresponsible words and actions of some schools(P1-3); Some school briefings avoid bullying to downplay responsibility(P1-4)
	It’s not difficult to fulfill responsibility in bullying governance (P2-3)	17	The facts of school bullying are clear(P1-5); There are commonalities in the characteristics of the bullies(P1-6)
**Difficulty in reconciling the interests of all parties calls for the fulfillment of responsibilities** **(P3-2)**	The victims and their family members defend their legal rights(P2-4)	27	Victims and family members have interest demands(P1-7); Victims and family members are dissatisfied with disposal results(P1-8); Victims and family members disagree with teacher(P1-9)
	The bullies and their family members defend their positions(P2-5)	20	The bullies deny bullying(P1-10); Attitudes among families of the bullies are inconsistent (P1-11)
	Teachers seek self-interest (P2-6)	11	Teachers make demands on the bullies and their families(P1-12); Teachers ignore or suppress claims of the victims and families(P1-13); Teachers want the incident to remain undisclosed(P1-14)
**School bullying governance is difficult and it is urgent to fulfill responsibilities** **(P3-3)**	Widespread negative impact of school bullying necessitates the fulfillment of responsibilities(P2-7)	68	Unfavorable to the victims(P1-15); Unfavorable to the family of the victims (P1-16); Disadvantageous to the bullies(P1-17); Disadvantageous to families of the bullies(P1-18); Unfavorable to other students(P1-19); Not conducive to teachers’ development(P1-20)
	The complexity of the governance environment requires the fulfillment of responsibilities(P2-8)	138	Families of the victims can’t cope with school bullying(P1-21); The victims conceal bullying(P1-22); Objective factors constraining governance(P1-23); Bystander and informed indifference(P1-24); Diverse ways to spot bullying(P1-25); Content or course of bullying is varied (P1-26); Bullying is complex(P1-27); Other parents’ attitudes vary(P1-28); School bullying is difficult to define (P1-29); Outdated concepts of governance(P1-30)
**Loopholes in laws and policies impede the fulfillment of responsibilities** **(P3-4)**	The binding force of current laws is weak (P2-9)	24	Unsound law(P1-31); Imperfect system(P1-32); Non-standardized criteria for judging bullying(P1-33)
	Difficulty in standardizing the autonomous definition of the incidents by teachers (P2-10)	17	Teachers deny the occurrence of bullying(P1-34); The review conclusions of education administrative department supports the investigation of teachers(P1-35)
	Teachers did not raise objections to the views of leadership or school (P2-11)	4	Teachers disagree with principals but without demur(P1-36); The class teacher has different views from school but remains silent(P1-37)
	Difficulty for other governing subjects to rectify the negligence of some teachers(P2-12)	10	Public security departments can’t influence the definition of some bullying incidents(P1-38); Hospital diagnoses have less impact on the definition of bullying incidents(P1-39)
**The facilitating role of the bullied seeking solutions to bullying(P3-5)**	The victims and their families seek reasonable treatment of the incident (P2-13)	18	Communicate with the bully or his/her family(P1-40); Communicate with teachers or school leaders(P1-41); Seek help from the police(P1-42); Looking for the victims(P1-43); Responding to outside questions(P1-44); Exposure of incidents through the media(P1-45)
	The families of victims questioned the disposition of the incident(P2-14)	24	Disposal of incident is contrary to the evidence of family(P1-46); Questioning the definition of the incident(P1-47); Question the disposition of incidents(P1-48); Question the review of incidents(P1-49)
**The media and the public play a supervision role(P3-6)**	Teachers and schools act actively after bullying exposure(P2-15)	46	Teachers act fast after the incidents come to light(P1-50); Teachers respond positively(P1-51); Teachers struggle to resolve the incident after exposure(P1-52)
	The local government acts actively after the incident come to light (P2-16)	30	Local education administrations respond positively and take a stand after the incident(P1-53); Local party committees and governments actively state and respond(P1-54); Local governments actively deal with incidents(P1-55)
	The media and the public are widely concerned about bullying(P2-17)	55	Incident sparks media and public debate(P1-56); Media and public opinion create pressure on govern(P1-57)
**Superior leaders and local government departments attaching importance to bullying helps schools fulfill their responsibilities (P3-7)**	Teachers take positive action after education administration’s intervention(P2-18)	7	Municipal Education Commission inquires about the incident(P1-58); Teachers dispose the incident quickly after the county education bureau makes instructions on the incidents(P1-59); Teachers act fast after county education bureau steps in(P1-60)
	Teachers perform positively after the intervention of local party committees and local government(P2-19)	5	Municipal Party Committee and Municipal Government attach importance to incidents(P1-61); County party committee and county government give instructions (P1-62); Leaders of town party committee promotes the fulfillment of teachers’ responsibility(P1-63)
**National laws and policies provide the basis for compliance** **(P3-8)**	Clarify requirements for fulfilling governance responsibilities(P2-20)	7	Setting criteria for identifying school bullying(P1-64); Requirements for teachers to fulfill their responsibility in preventing school bullying(P1-65)
	Accountability of persons not fulfilling their responsibilities (P2-21)	8	Accountability of school staff(P1-66); Accountability of other responsible persons(P1-67)

### 5.3 Theoretical saturation and reliability tests

The theoretical saturation test refers to the verification of the saturation of the main category codes in the research findings by testing whether new main category codes can be obtained from the textual materials. In this paper, the systematic coding of the 101 textual materials in the remaining 19 cases did not result in new significant categories, and the logical relationships between categories remained unchanged, so the subcategories and main categories obtained in this paper passed the theoretical saturation test.

In order to ensure the independence and reliability of the two coding sessions, this paper recoded the textual data one week after the first coding session and placed the second result in the group “LYY coding”. The second coding was still done on the 215 data of the first 36 cases. Unlike the first coding, in this process of coding, each case text was passed through open coding, spindle coding, and selective coding, and new concepts and categories were added gradually to complete the coding of the 36 cases to obtain the final master category system. Subsequently, the textual data of the remaining 19 cases were compared and passed the theoretical saturation test. The “Code Comparison” function in NVivo11 tested the coding reliability by comparing the percentage of coding agreement between the two clusters. In the test data, the lowest consistency value was 78.95% (only one item) and the highest was 100%, and the Kappa coefficients of the comparative codes were above 0.8, which indicated that the codes in this paper maintained a very high overall consistency, i.e., a high level of confidence.

## 6 Model construction and cause analysis of influencing factors

**Fig 5:**
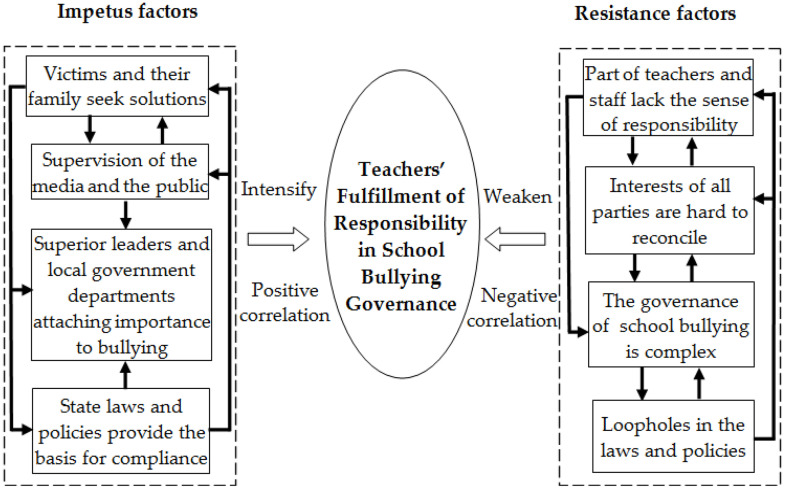
The “two-factors model” that influences teachers’ fulfilling of responsibility.

The main category, subcategory and initial category obtained from coding and analysis of the typical cases of school bullying can encapsulate the factors affecting teachers in fulfilling their responsibility of bullying governance. By constructing the influencing factor model, we can further explore the optimization path for the teachers to fulfill responsibilities through the model. In the process of school bullying governance, teachers’ fulfillment of their responsibilities is affected by a variety of factors, which can be divided into impetus factors and resistance factors, thereby forming a “two-factors model” (Fig 5). One or several impetus factors can enhance the teachers’ fulfillment of their responsibilities, while single or multiple resistance factors can weaken the teachers’ fulfillment of their responsibilities.

### 6.1 Model construction of influencing factors

#### 6.1.1 Impetus factors for teachers to fulfill responsibilities.

The impetus factors in the “two-factors model” are the elements that promote teachers’ effective accountability, including the bullied party seeking to solve the problem (P3-5), the media and the public playing a supervision role (P3-6), local government leaders and departments attaching importance to school bullying (P3-7), and the state enacting laws and policies to provide a basis for accountability (P3-8). The bullied party seeking solutions to the school bullying incident is an important factor in promoting teachers’ fulfillment of responsibility. It is precisely due to the valid doubts raised by the bullied and their family members regarding the teachers’ and education administration’s definition of the incident and measures (P2-14) through communication and exposure (P1-40, etc.) that many incidents of school bullying were addressed in a more reasonable manner (P2-13), which facilitated the effective fulfillment of teachers’ responsibility. Supervision of the media and the public can strengthen teachers’ responsibilities, since the widespread attention from media and public (P2-17) exerts public opinion pressure on teachers, prompting them to actively handle incidents after exposure (P2-15, P2-16). Leadership and supervision of school bullying governance by local party committees and people’s governments at all levels facilitates teachers’ fulfillment of their responsibilities. The instructions from leaders of local party committees and people’s governments can urge teachers to actively and effectively manage school bullying (P2-19). The intervention of education administrative departments promotes very proactive behavior from teachers (P2-18). The state has formulated laws and policies to clarify the requirements of governance responsibilities for teachers (P2-20), and the accountability of school staff and other responsible persons who fail to fulfill their responsibilities (P1-68, P1-69). This encourages them to actively fulfill their responsibilities.

#### 6.1.2 Resistance factors for teachers to fulfill their responsibilities.

The resistance factors in the “two-factors model” are those that prevent teachers from effectively fulfilling their responsibilities, including the lack of a sense of responsibility on the part of staff (P3-1), the difficulty in coordinating the interests of various parties (P3-2), the difficulty in school bullying governance (P3-3), and the loopholes of some parts of the laws and policies (P3-4). The lack of the sense of responsibility on the part of some faculty members is the most important factor hindering the fulfillment of responsibility. Irresponsible words and actions (P2-1, P2-2) indicate that their lack of attention to school bullying or their weak will to govern, which will inevitably weaken the sense of responsibility. In many cases, it is not difficult to fulfill the responsibility of school bullying governance (P2-3), as most bullying incidents at school are straightforward(P1-5), the bullies share some characteristics (P1-6), and the governance personnel can improve the effectiveness of school bullying governance as long as they have a strong sense of responsibility. In the process of school bullying governance, the difficulty in coordinating the interests of all parties is not conducive to the fulfillment of the teachers’ responsibilities. The victims, who have suffered physical and mental injuries, and their families must defend their legitimate rights and interests (P2-4), while the bullies and their families often try their best to deny the school bullying incidents in order to evade disciplinary actions, compensation, or the stigma of being labeled as “school bullies” (P2-5). For the sake of their own reputation, teachers may prefer to solve the incident privately (P1-14), advocate for their own demands (P1-12), overlook or downplay the needs of the bullied party (P1-13). When the interests of all parties are difficult to be reconciled, teachers cannot fulfill their responsibilities of governance. Moreover, the complexity of school bullying makes it more challenging for teachers to fulfill their responsibilities. School bullying incidents have negative impacts on the bullies, the victims, their families, and teachers (P1-15, P1-16, etc.), which lead to a more complex governance environment (P2-8). Teachers are required to fulfill their responsibilities effectively in order to better manage the problem of school bullying. The defects in laws and policies may undermine teachers’ fulfillment of their responsibility in school bullying governance, because weakly binding laws and policies (P2-9) complicate teacher’s characterization of the incident (P2-10). Imperfect laws and policies suppress the motivation of teachers and other governance subjects. They will not challenge the words and actions of the leaders or the departments, even if the words and actions are inappropriate (P2-11). In some cases of bullying, despite having evidence, the public security organs or hospitals find it difficult to influence the characterization of school bullying incident (P1-38, P1-39).

To summarize, in the process of school bullying governance, the factors affecting teachers’ fulfillment of their responsibilities include not only the teachers themselves, but also the parties involved in bullying, the media and the public, as well as external factors such as laws and policies. In the “two-factors model”, the impetus factors have a positive correlation with the fulfillment of responsibilities, and the resistance factors are negatively related to the fulfillment of responsibilities. The correlation between impetus factors and resistance factors is inverse; the strengthening of one leads to the weakening of the other.

The augmentation of impetus factors helps to reduce the influence of resistance factors, thus facilitating the fulfillment of teachers’ governance responsibilities. Conversely, a rise in resistance factors will weaken the effect of impetus factors, posing challenges to the effective execution of governance responsibilities. In order to strengthen teachers’ fulfillment of their responsibilities, China has to vigorously enhance the impetus factors in the “two-factors model”, while at the same time minimizing the resistance factors.

### 6.2 Impact mechanism of influencing factors

Why do these factors influence teachers’ fulfillment of their responsibilities in school bullying governance? As typical street-bureaucrats, the choice of teachers’ behavior is consistent with the content of Street-Level Bureaucracy Theory. Therefore, this paper analyzes the reasons why these factors influence teachers’ fulfillment of their responsibilities in school bullying governance through Street-Level Bureaucracy Theory, thereby laying the foundation for further investigations into optimization paths.

#### 6.2.1 Impact mechanism behind impetus factors.

Among the impetus factors for teachers to fulfill their responsibilities, the bullied and their families are the key force to push teachers to fulfill responsibilities in school bullying governance. If teachers fail to deal with school bullying properly, it means that teachers as street-level bureaucrats are unable to provide public services such as fair education to the bullied. In order to safeguard their legitimate rights and interests, the bullied and their families may persistently express their reasonable demands to teachers, and actively seek for a proper resolution of the incident. Faced with the reasonable requests of the bullied and their families, teachers with governance responsibilities cannot evade them. They are obligated to address these concerns fairly and reasonably, thereby urging teachers to deal with school bullying incidents in a fair and just manner. This in turn motivates teachers to actively fulfill their governance responsibilities. Victims and their families seeking to solve the incident also attract the attention and supervision of the media and the public, and even the attention of the leaders or relevant departments in local government. This will push the state to formulate relevant laws and policies to strengthen the fulfillment of the responsibility of teachers. Next, teachers, as street-level bureaucrats, have a great deal of discretion, making it difficult to supervise them and hold them accountable for the definition and disposition of school bullying incidents. If teachers’ misconduct in the governance of campus bullying can be brought to light, and they are open to the supervision, questioning and feedback of public opinion, teachers will mind their behavior and tend to fulfill their responsibilities. The media is called the “the fourth power” in parallel with the legislative power, executive power and judicial power, which can timely reveal teachers’ shortcomings in the bullying governance, and better supervise teachers in fulfilling their responsibilities. School bullying is a phenomenon that can easily provoke social nerves. The social debate triggered by the heightened public concern will also exert external pressure on teachers to fulfill their responsibilities and regulate them to effectively fulfill their governance responsibilities. The scrutiny and oversight of the media and the public can often prompt local government leaders or departments to attach importance to school bullying, which may push teachers to fulfill their responsibilities actively. Furthermore, superior leaders and government administrations can use their dependency and gaming relationship with teachers to promote teachers’ fulfillment of their responsibilities in school bullying governance. In China, school bullying governance is led and supervised by the government, who will affect the allocation of some of teachers’ work resources. Teachers need to accept the leadership and supervision of the government. The attention of local government leaders or competent departments at all levels can reinforce teachers’ fulfillment of their responsibilities in the prevention, management and resolution of school bullying. Especially after the occurrence of school bullying incidents, the instructions of local government leaders on the governance of the incident, along with the participation of education, public security and other departments, will urge teachers to actively assume their roles in school bullying governance. Finally, to regulate teachers’ discretion, monitor and hold teachers accountable for their behavior, it is important to enact laws and make policies. Laws are mandatory, and policies have guiding and controlling functions. The clear obligations of teachers stipulated by the state’s laws and policies can strengthen teachers’ active fulfillment of their responsibility in the governance of school bullying. These legal and policy requirements encourage local government leaders or departments to pay more attention to the governance of school bullying, offer institutional support for the media and the public to monitor the responsibilities of teachers, and provide a legal basis for victims and their families to seek appropriate solutions to the incidents.

#### 6.2.2 Impact mechanism behind resistance factors.

Despite the fact that Chinese laws and policies have clearly defined school bullying and stipulated the contents of teachers’ responsibilities, some teachers lack a sense of responsibility. They still describe school bullying as a “joke”, “horseplay” or an experience in growing up in order to avoid their responsibilities. This is because teachers have discretion in the governance of school bullying, are directly involved in its characterization and disposition, and are difficult to second-guess and hold accountable for the appropriateness of their behavior. Teachers have a lot of work to do. Apart from teaching, they also need to prepare lessons, examinations, respond to inspections by superiors and ensure school safety. These tasks increase the workload of teachers, who then try to minimize the content of their work or debase the quality of their work. Moreover, teachers who are street-level bureaucrats treat different students with favoritism or discrimination, and some students may not be treated fairly, which is not conducive to teachers’ fulfillment of their responsibilities in the governance of school bullying. Some teachers fear a tarnished reputation when fulfilling their governance responsibility [50], and some class teachers may know that students are being bullied but do nothing. Teachers’ passivity and lack of competencies to deal with school bullying add to the difficulty in performing duties [51]. The lack of a sense of responsibility on the part of teachers hinders their management of school bullying, and may lead to difficulties in coordinating the interests of all parties and further complicate the problem of school bullying. Teachers need to strengthen their own sense of responsibilities and be attentive, careful, and patient in their daily teaching and management so as to strengthen their governance responsibilities. What’s more, all parties of bullying governance have their own interests, which can sometimes lead to conflicts among different parties. Street-level bureaucrats may adopt some strategies to reduce the quantity and quality of public services. As street-level bureaucrats, teachers may protect their own interests by reducing their bullying governance responsibilities for students and parents, and this can result in conflicts of interest. When the interests of all parties are hard to coordinate, the difficulty of school bullying governance increases. Teachers then need to devote more time, energy, knowledge and ability, which may cause them to be perfunctory. The more challenging it is to coordinate these interests, the more likely the teaching staff is to lack a sense of responsibility. Besides, the complex and varied work situations faced by street-level bureaucrats make governance more difficult. The inherent complexity of school bullying, including a wide variety of times, locations, bullies, the bullied, causes and other aspects, makes it more difficult for teachers to fulfill their responsibilities. The complexity of school bullying increases the difficulty for schools to fulfill their responsibilities as well. School bullying often occurs in locations without adult supervision, where the bullies ensure there are no adults present before they bully someone [52]. This leads to the concealment of school bullying, which increases the complexity of governance and is not conducive to the fulfillment of teachers’ responsibilities. School bullying occurs in a variety of places, such as classroom, corridor, bathroom, playground, dormitory, school gate, and so on. Meanwhile, the reasons of school bullying are diverse. School bullying may be caused by appearance, size, weight, outsider identity, religious beliefs, sexual orientation, wealth or poverty, and so on. Faced with such complex and changeable school bullying incidents, many teachers undertaking teaching, testing, administration and other tasks may shirk their responsibilities as they are unable to deal with bullying effectively. The complexity of school bullying may also lead to difficulties in coordinating the interests of various parties and the need to improve laws and policies. Finally, while laws and policies can regulate street-level bureaucrats’ fulfillment of their responsibilities through supervision and accountability, their effectiveness is not ideal and needs continuous improvement. The leak of laws and policies may make some teachers to have a fluke mentality, fail to comply with the relevant regulations, or lose the motivation to fulfill their responsibilities due to a perceived inability to achieve the expected goals, thus weakening the teachers’ sense of responsibilities. Imperfect laws and policies on the governance of school bullying not only do nothing to bolster the school’s sense of responsibility, but also do not help to coordinate the interests of all parties, exacerbating the already complex problem of school bullying.

## 7 Optimization paths for schools to effectively fulfill responsibilities

Effective fulfillment of responsibilities by teachers is an important guarantee for China to achieve the goal of good governance in school bullying. Previously, China has adopted the methods of proactive advocacy and post-event punitive accountability to urge teachers to actively fulfill their responsibility in the governance of school bullying. While this approach yielded certain results, it also exposed some problems of ineffective fulfillment of responsibility. Inspired by the Street-Level Bureaucracy Theory, in order to promote the effective fulfillment of teachers’ responsibilities, China needs to optimize the following paths to enhance the impetus and eliminate the resistance.

### 7.1 Perfecting the system

A sound system can regulate the behavior of street-level bureaucrats, such as teachers, by clarifying responsibilities, supervision, and accountability. Many scholars endorse the idea that legislation should impose specific legal obligations on teachers and make them take responsibility for bullying behavior they are aware of or should reasonably be aware of, which would objectively bring a larger proportion of bullying behavior into the purview of statutory law regulation [53]. China has enacted a number of laws and policies to address school bullying, effectively safeguarding the physical and mental health and legal rights of students who are bullied in school bullying. The case study of school bullying suggests that the current system in China needs to be improved in order to enhance the impetus and reduce resistance for teachers to fulfill their responsibilities.

#### 7.1.1 Establishing a system for ex-ante and in-process error correction.

At present, China urges teachers to fulfill responsibilities through the post-event accountability system. However, there’s a lack of timely correction for the dereliction of duty in teachers’ words and deeds, both prior to and during incidents. The ex-ante and in-process correction system should be jointly participated by multiple subjects such as schools, education administrative departments, public security organs, health departments, hospitals, representatives of parents, experts, etc. In this system, the rights and responsibilities among subjects are equal, important matters such as the definition of bullying incidents are democratically consulted, different views are allowed to exist, and a good atmosphere for correcting deviation is created, thereby enabling the timely correction of irresponsible words and deeds of teachers in the stage of prevention and disposal.

#### 7.1.2 Perfecting the accountability system.

The responsibilities of teachers need to be detailed so that the governance responsibilities are assigned to specific positions and personnel, thereby aligning the work content and personnel responsibility. The accountability system should incorporate a mechanism for retrospective examination and lifelong accountability, preventing school staff from slack and perfunctory performance in the fulfillment of responsibilities.

#### 7.1.3 Improving information disclosure system.

The effectiveness of accountability and public scrutiny will hardly be realized if information is disclosed in an inappropriate form and lacks details. On the premise of protecting the privacy of both parties involved in bullying, publicizing information on governance is conducive to promoting teachers’ active fulfillment of their responsibilities. Information disclosure needs to be prompt in order to quickly gain the upper hand in public opinion, curb rumors, and grasp the initiative of governance. The content of the information disclosed must be accurate, objective, true, authoritative and neutral. The investigation basis, the characterization criteria, the disposal process, the aftermath of the initiative, and the participants involved in the incident should be made public to accept social supervision.

### 7.2 Strengthening publicity, education and training

In order to motivate street-level bureaucrats to better provide quality public goods or services, it is necessary to change their mindset through measures such as publicity, education and training. Publicity, education and training help to change the governance mindset of teachers and motivate them to change their behavior and then actively fulfill their responsibilities. In the governance of school bullying, training teachers in identifying such incidents is considered a priority, providing them with guidelines for action [54]. Publicity and education have a similar effect. China has begun to carry out publicity, education and training for teachers. However, the performance of some staff in the school bullying cases indicates that the publicity, education, and training need to be strengthened, so that teachers may acquire scientific knowledge of school bullying governance, establish correct concepts of governance, reach a consensus on the incident definition, responsibility division, and the disposal method, and reduce differences among all parties. Publicity, education and training play a significant role in encouraging teachers to pay more attention to school bullying governance, thus increasing the motivation of teachers to fulfill their responsibilities and reducing resistance.

#### 7.2.1 Making the content comprehensive.

The content of publicity, education and training should be comprehensive. Publicity, education and training should enable school staff to fully understand the current situation of school bullying governance, and familiarize themselves with the laws and policies on bullying governance. Teachers have to be acquainted with the characteristics, causes, governance dilemmas, countermeasures and other professional knowledge of school bullying, so as to rectify their attitudes towards school bullying governance, clarify their own responsibilities, and pay more attention to school bullying governance, thus enhancing the fulfillment of responsibilities.

#### 7.2.2 Normalizing the time.

For the sake of better fulfilling responsibilities, it is necessary to normalize the time of publicity, education and training. Campaign governance is a kind of governance method that adopts unconventional, non-institutional and non-professional means to quickly achieve certain effects. China’s current publicity, education and training are inclined to be the campaign mode, which lacks continuity. However, the governance of school bullying is a long-lasting battle, and publicity, education and training must be normalized. China should formulate a long-term plan to integrate school bullying governance into the daily work and subconsciousness of school staff through regular publicity, education and training, so as to form professional ethics and strengthen the fulfillment of responsibilities.

#### 7.2.3 Diversifying the approaches.

In order to obtain good results, the approaches of publicity, education and training are better to be diverse. Omnimedia conveys information through different senses, presenting it with the help of newspapers, television, radio, and the Internet, and utilizing a variety of forms, such as text, audio, video, and pictures. Chinese publicity combines hard and soft publicity to maintain its authority, market relevance and credibility [55]. The same is true for education and training. Nowadays, smartphones and the Internet have become very popular. It is a good way to conduct publicity, education and training through microblog, WeChat, QQ, “Tik Tok” and other Mobile client application.

### 7.3 Reinforcing supervision of the teachers

Despite the less than optimal results, supervision remains an important way to urge street-level bureaucrats to fulfill their responsibilities. From the coding analysis of bullying cases, it can be seen that reinforcing supervision of the local government, the media and the public on teachers is an important impetus factor to promote teachers to effectively fulfill their responsibility of school bullying governance. At the same time, reinforcing supervision can alleviate the resistance factors and enhance the sense of responsibility of teachers.

#### 7.3.1 Strengthening the supervision from the government, media, and the public.

Local party committees and people’s governments, especially the education administrative departments, provide leadership and constraints on teachers. At the same time, the media and the public can urge the school staff to correct their improper words and deeds. This multi-pronged approach to supervision can ensure teachers’ fulfillment of their responsibility for school bullying governance. By reinforcing the constraints and boundaries on teachers’ words and actions, the local government, the media and the public can reduce the likelihood of teachers attempting to shirk their responsibilities, plug the loopholes in governance, prevent school bullying governance from the influence of black-box operations and interpersonal networks, and urge teachers to fulfill their responsibilities for dealing with school bullying incidents in a fair and just manner.

#### 7.3.2 Providing guarantees for the supervision.

China needs to develop a system to ensure that the government, media and public can oversee teachers in fulfilling their responsibilities of school bullying governance. No unit or individual can try to cover up the truth about school bullying incidents. Take media as an example, if the media cannot withstand the pressure from capital and power, it cannot perform their supervisory functions [56]. China should avoid artificial obstacles to supervision, prevent individuals from obstructing the reporting of incidents, and eliminate behavior such as the deletion of posts by online media.

#### 7.3.3 Regulating the behavior of supervision.

Supervision is a “double-edged sword”. When applied properly, it can promote the school bullying governance. However, if not implemented carefully, it can also hinder the governance of school bullying. This is because some local government officials choose to deny school bullying incidents and downplay the responsibility of teachers in order to maintain the image and reputation of local education and society. Third-party supervisory organizations have more professional knowledge, technology, capital, and human resources, so the government can collaborate with the third parties to supervise [57], promoting teachers to fulfill their responsibilities. In addition, media reports are characterized by complex perspectives and multiple orientations. The media’s subjective desire to report truthfully and objectively may at times conflict with their wishes, and they may also form public opinion noise or even deviate from the incidents themselves. This is also a feature of public supervision. These drawbacks are not conducive to the governance of school bullying. Therefore, while strengthening the supervision of teachers by the local government, the media and the public, China needs to regulate them to avoid damaging the fairness of governance.

### 7.4 Improving the overall governance environment

The environment has a large influence on human behavior. Improving the overall governance environment can enhance the awareness of street-level bureaucrats in providing public goods and services, standardize the use of discretion, and proactively fulfill their governance responsibilities. When the external environment is not supportive, some teachers may aspire to fulfill their responsibilities, but find it difficult to achieve the expected results [58]. The case coding and the “two-factors model” in Fig 5 show that the factors affecting teachers’ fulfillment of responsibilities are influenced by external conditions such as governance environment, and improving the overall governance environment is conducive to promoting the fulfillment of responsibilities. On the one hand, other governance subjects should intensify their fulfillment of responsibilities. Although China requires schools and 11 departments to participate in the governance of school bullying, when it comes to fulfilling their responsibilities, schools, education administrative departments and public security departments are the main players, while other governance subjects are reduced to supporting roles. In the future, other governance subjects in China need to intensify their efforts in fulfilling their responsibilities, preventing teachers from acting arbitrarily and compelling them to effectively uphold their duties. Each governance subject must change from fragmented management to holistic governance, strengthen the sharing of information and other resources, and enhance the coordination among governance subjects. On the other hand, existing studies have shown that school bullying is often influenced by bullying politics, economy, culture, etc. [59]. It is the result of a combination of environmental factors such as family, school, and society [60]. Tackling school bullying, including cyberbullying, is not the responsibility of teachers alone, but a collective responsibility of the entire educational community, in addition to the media [61]. Unfavorable environments that lead to high frequency of school bullying will increase the governance burden of teachers and backfire on the fulfillment of their responsibilities. Therefore, improving the overall governance environment can reduce the occurrence of school bullying and help teachers to fulfill their responsibilities. Family environments that promote disdain for the poor and admiration for the rich, social phenomena of bullying, and movie and TV episodes of bullying may all subconsciously breed the “tumor” of school bullying. Consequently, China needs to optimize the family education model, rectify the public security environment, eliminate social evils, purify the cultural atmosphere, strengthen fairness and justice, so as to remove the breeding ground of school bullying from the aspects of ideology, culture and safety, reduce the difficulty of school bullying governance, and encourage teachers to fulfill their responsibilities more actively, proactively and confidently.

## 8 Discussion

### 8.1 Heterogeneity analysis

According to the analysis of the “two-factors model”, teachers may be driven by multiple impetus factors or hindered by different resistance factors when fulfilling their responsibilities in the governance of school bullying. This is a holistic study based on a group of grassroots teachers. In the process of school bullying governance, because of differences in cognition, knowledge, experience, ability, energy and other aspects, different teachers are heterogeneous in the face of impetus factors and resistance factors. In terms of the impetus factors, if a teacher supports the bullied students and their family members, is willing to accept media and public supervision, obeys the leadership of the local government or higher-level departments, and abides by the provisions of national laws and policies, this can form a motivation to promote the fulfillment of responsibilities. In governance practice, when individual teachers do not support the bullied and violate the provisions of laws and policies, these impetus factors cannot play a promoting role. When it comes to the resistance factors, not all teachers lack a sense of responsibility, and some teachers are still able to perform their duties wholeheartedly to deal with school bullying. Although the interests of different subjects are difficult to coordinate, the problem of school bullying is complex, and there are loopholes in laws and policies, if teachers are able to deal with school bullying incidents in a fair and impartial manner, the adverse impact of resistance factors can be mitigated to a certain extent. Therefore, whether it is an impetus factor or a resistance factor, there is heterogeneity among different teachers in fulfilling their responsibilities of school bullying governance.

### 8.2 Future research direction

Future research direction: This paper explored the responsibility fulfillment of grassroots teachers in the governance of school bullying. In the future, it is necessary to study the responsibility fulfillment of middle-level and high-level school staff, analyze the influencing factors of their fulfillment of responsibilities, and compare whether there are any differences in the influencing factors and mechanisms between grassroots teachers and middle-level and high-level ones. Moreover, it’s worthwhile to discuss the interaction between high-level school personnel such as the principals and the government, parents, and the society, how to better fulfill their responsibilities, and how to supervise and restrain the behavior of these faculty members. These are the contents that need to be studied in the future.

### 8.3 Limitation

This paper focused on the influencing factors of teachers in the governance of school bullying, and constructed a “two-factor” model including impetus factors and resistance factors. The findings have certain reference value, but there are still limitations. On the one hand, the analysis materials in this paper were compiled from school bullying incidents reported by schools, government and media. As these are second-hand data, rather than first-hand direct materials, there are certain deficiencies. On the other hand, based on the framework of Street-Level Bureaucracy Theory, this paper analyzed the impetus factors and resistance factors that affect teachers’ fulfillment of their responsibilities, and put forward optimization countermeasures according to the analysis results. However, the discussion did not delve into the influencing factors beyond the theoretical content of street-level bureaucracy, such as teachers’ psychology, knowledge, and ability in the process of campus bullying governance, which also affect the fulfillment of responsibilities to a certain extent.

## 9 Conclusion

Effective fulfillment of teachers’ responsibilities is the key to the governance of school bullying. Although China has enacted laws and policies to clarify the responsibilities of teachers, after analyzing the behavior of school staff in school bullying cases, it was found that some teachers and school leaders still failed to fulfill their responsibilities actively. According to the Grounded Theory, with the help of NVivo11 qualitative analysis software, this paper coded the textual data of 55 cases of school bullying, summarized the influencing factors on teachers’ fulfillment of their responsibilities, and constructed a model of “two factors” including impetus and resistance. In the light of the Street-Level Bureaucracy Theory, it was also found that the impetus factors influencing teachers’ fulfillment of their responsibilities in managing school bullying include: the efforts of the bullied party to find a proper solution to the problem, the supervision role of the media and the public, the importance attached by leaders and departments such as police in the local government, and the basis for fulfilling the responsibilities provided by the state laws and policies. Meanwhile, there are also factors that prevent teachers from fulfilling their responsibilities, such as the lack of responsibility on part of teachers, the difficulty of coordinating the interests of different governance subjects, the complexity of school bullying itself, and the deficiencies of some legal and policy content. In the process of school bullying governance, impetus factors will enhance teachers’ fulfillment of their governance responsibilities, while resistance factors will weaken teachers’ fulfillment of their governance responsibilities.

In order to effectively encourage teachers to fulfill their responsibilities of bullying governance, China needs to enhance impetus factors and eliminate resistance factors. According to the Street-Level Bureaucracy Theory, China needs to change the behavior of teachers and optimize the current paths for them to fulfill the responsibilities of school bullying governance. It should improve the laws and policies governing school bullying, strengthen the publicity, education and training of school teachers, heighten the supervision of the government, the media, and the public on teachers, and improve the overall environment of bullying governance, so as to enhance the sense of responsibility of teachers and urge them to assume the responsibility of bullying governance. By now, these optimization paths are external forces acting upon teachers. In the future, how to transform the external guidance and constraints on teachers into their internal sense of responsibility, and intrinsically motivate them to fulfill their responsibilities for the governance of school bullying is a matter to be further explored. This will help achieve better results in governance, build a safe and harmonious campus environment free of bullying for students, and promote the efficient and sustainable development of education.
